# Transcription of Click-Linked DNA in Human Cells**

**DOI:** 10.1002/anie.201308691

**Published:** 2014-01-22

**Authors:** Charles N Birts, A Pia Sanzone, Afaf H El-Sagheer, Jeremy P Blaydes, Tom Brown, Ali Tavassoli

**Affiliations:** Chemistry, University of SouthamptonSouthampton, SO17 1BJ (UK); Cancer Sciences, Faculty of Medicine, University of SouthamptonSouthampton, SO16 6YD (UK); Dept. of Science and MathematicsSuez University, Suez, 43721 (Egypt); Department of Chemistry, University of Oxford, Chemistry Research Laboratory, OxfordOX1 3TA (UK)

**Keywords:** click chemistry, DNA ligation, gene technology, nucleic acids, synthetic biology

## Abstract

Click DNA ligation promises an alternative to the current enzymatic approaches for DNA assembly, with the ultimate goal of using efficient chemical reactions for the total chemical synthesis and assembly of genes and genomes. Such an approach would enable the incorporation of various chemically modified bases throughout long stretches of DNA, a feat not possible with current polymerase-based methods. An unequivocal requirement for this approach is the biocompatibility of the resulting triazole-linked DNA. The correct function of this unnatural DNA linker in human cells is demonstrated here by using a click-linked gene encoding the fluorescent protein mCherry. Reverse transcription of mRNA isolated from these cells and subsequent sequencing of the mCherry cDNA shows error-free transcription. Nucleotide excision repair (NER) is shown to not play a role in the observed biocompatibility by using a NER-deficient human cell line. This is the first example of a non-natural DNA linker being functional in a eukaryotic cell.

To date, there has been no report of an unnatural DNA-backbone linker[1,2] that is functional in human cells (or other eukaryotic cells). Such a linker would be significant for several reasons; first, it would open up the possibility of the purely chemical synthesis and assembly of heavily modified genes and genomes, which would enable informative experiments in cell biology. Second, it would illustrate that the cellular machinery tolerates variations in the backbone of canonical DNA, which would have a significant impact on current approaches to the assembly of large DNA fragments; no longer bound by the need for a phosphodiester linker, chemists would be free to explore and develop more efficient chemical DNA-ligation reactions. Third, the self-templating property of DNA, combined with a highly efficient chemical ligation, would potentially allow one-pot gene synthesis. We have recently reported click DNA ligation: the use of copper-catalyzed alkyne–azide cycloaddition (CuAAC) for joining DNA strands (Figure 1 a)[1] and the biocompatibility of the resulting triazole-linked DNA in *Escherichia coli.*[1,3] We next sought to probe the biocompatibility of click-ligated DNA in human cells.

**Figure 1:**
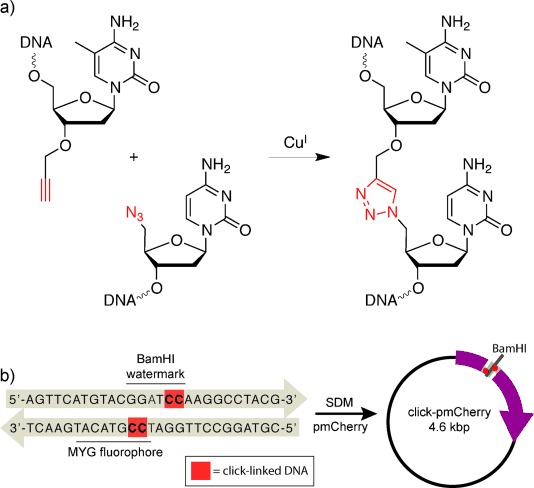
Assembly of the click-linked pmCherry plasmid. a) Oligonucleotides functionalized with a 5′ azide are ligated to oligonucleotides with a 3′ alkyne through the CuAAC reaction, thereby resulting in a triazole backbone linker. 5-Methylcytosine (5-MedC) was used as the 5′ nucleobase of the C–triazole–C linkage for synthetic convenience; the 3′ propargyl-5-MedC is derived from thymidine. b) The click-linked primers used to construct the click-linked pmCherry plasmid by SDM contain a readily identifiable BamHI watermark.

An optimized site-directed mutagenesis (SDM) protocol[3] was used to construct a plasmid containing a click-linked gene encoding the fluorescent protein mCherry.[4] Forward and reverse mutagenic primers, each containing a pair of triazole-linked cytosine nucleosides, were designed to overlap in the region encoding the fluorophore of mCherry (Figure 1 b), which is formed from a tripeptide MYG motif (methionine 71, tyrosine 72, and glycine 73). These primers introduce triazole-linked cytosine dinucleotides into the forward and reverse strands of the mCherry gene, four base pairs apart, in a region critical for the fluorescence of mCherry (Figure 1 b). Correct transcription through the click-linked DNA would result in the cells displaying the red fluorescence associated with mCherry expression, whereas any errors in transcription arising from the presence of the artificial linkers would result in an easily identifiable, nonfluorescent phenotype. The click-linked primers were also designed to introduce a silent BamHI restriction site into the region encoding the MYG fluorophore (by altering the glycine codon from GGC to GGT). This restriction site acts as a watermark that allows rapid identification of the progeny of click-linked plasmids by restriction digestion analysis or sequencing. As previously demonstrated,[3] the SDM product only contains the click-linked gene (Figure S1 a, b). A canonical equivalent of the above plasmid was generated with normal primers that lacked both the artificial linker and the BamHI restriction site (GGC glycine codon not changed) for use as a positive control. The above SDM protocol was also repeated with water in the place of mutagenic primers and the resulting solution used as a negative control in the following experiments.

To probe the biocompatibility of triazole-linked DNA in human cells, the products of SDM with click-linked or normal mutagenic primers were dialyzed against water to remove buffer salts and microinjected into a breast cancer cell line (MCF-7). Microinjection allows single-cell analysis of mCherry expression from the click-linked DNA; injected MCF-7 cells are readily identified by the FITC–Dextran dye coinjected with the plasmid encoding mCherry. After incubation for 24 h, 90±4 % of the 100 MCF-7 cells injected (in the cytoplasm) with the template pmCherry plasmid displayed the red-fluorescent phenotype associated with mCherry expression (Figure 2 a, b). The SDM products contain a single-strand break that will need to be repaired prior to transcription,[5] hence the SDM products were injected into the nucleus of the MCF-7 cells rather than the cytoplasm. It should be noted that the repair of single-strand breaks does not involve nucleotide excision,[5] thus this process will not affect or alter the triazole DNA-backbone linker. Nuclear injection with the product of SDM with canonical primers resulted in 51±13 % of the 596 injected cells displaying the red fluorescence associated with mCherry expression. The same experiment repeated using the product of SDM with click-linked primers resulted in 42±12 % of the 553 injected cells showing the red fluorescence of mCherry expression (Figure 2 a, b). The lower percentage of cells expressing mCherry from the SDM products (canonical and click-linked) versus those with plasmid DNA is likely a consequence of using the SDM products directly rather than first subcloning into *E. coli* as is the norm (subcloning increases the concentration of the resulting plasmid but erases the artificial linker). These results demonstrate the biocompatibility of triazole-linked DNA and that it functions in human cells at similar levels to the canonical equivalent. It should be noted that the pmCherry plasmid, which contains an SV40 origin of replication, is not replicated in MCF-7 cells; these cells are not SV40 transformed[6] and hence do not express the SV40 large T antigen required for replication of the plasmid.[7] This precludes the possibility that transcription through click-linked DNA requires prior replication to remove the triazole-linked backbone.

**Figure 2:**
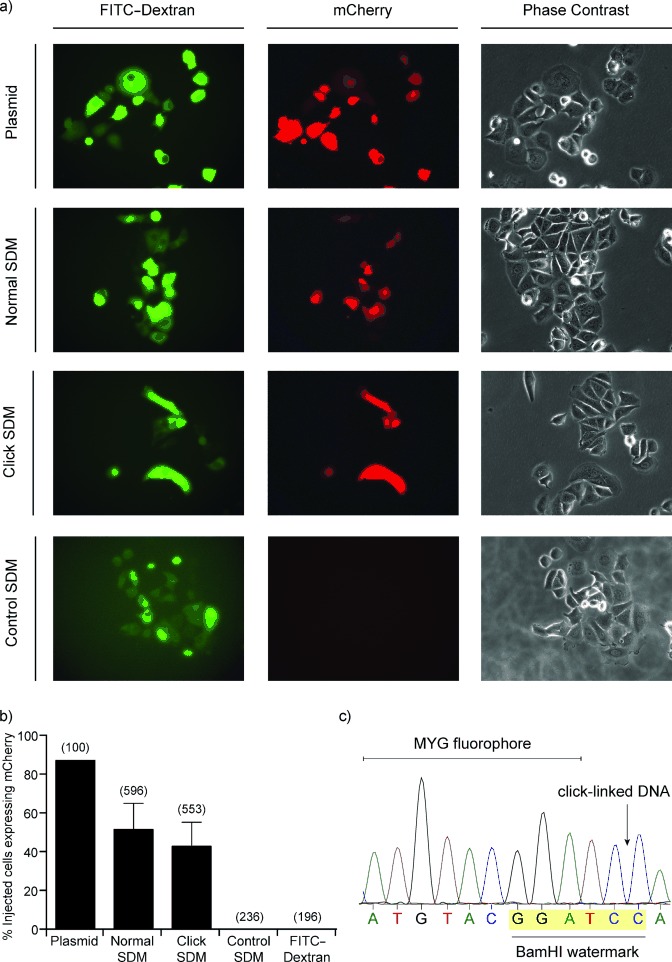
Click-linked DNA is functional in human cells. a) Representative images of microinjected MCF-7 cells. Cells were injected with the pmCherry plasmid or the products of SDM with canonical primers, click-linked mutagenic primers, or water SDM control. The injected cells are readily identifiable through the green fluorescence of the coinjected FITC–dextran dye. Those cells expressing mCherry from the injected plasmids can be identified by their red fluorescence. The phase contrast channel shows the population of MCF-7 cells. b) The percentage of injected cells that displayed the red fluorescent phenotype. The data is the mean of three independent repeats. The numbers in brackets above each bar indicate the total number of cells injected. c) mRNA was isolated from cells injected with click-linked pmCherry, reverse transcribed and sequenced. The BamHI watermark was present in all examined cases.

To assess the fidelity of transcription through the click-linked mCherry gene, mRNA was isolated from injected cells and reverse transcribed. The mCherry cDNA was amplified by PCR and cloned (by TA cloning) to enable the analysis of individual copies of the gene. The resulting plasmids were isolated from 25 colonies and sequenced. In every case, the BamHI watermark associated with the triazole DNA linker was present (Figure 2 c) and the mCherry gene did not contain any mutations. The plasmids from an additional 100 colonies were analyzed by restriction digestion for the presence of the BamHI watermark, which was found to be present in all cases (Figure S2), thus demonstrating their origin from the click-linked mCherry gene.

The role of nucleotide excision repair (NER) in the biocompatibility of click-linked DNA in mammalian cells was next probed. Xeroderma pigmentosum group A protein (XPA) is a key subunit of the nucleotide excision repair pathway in mammalian cells that recognizes the increased deformability of damaged sites,[8] and is absolutely required for NER.[9] We used an XPA-deficient cell line (XP2OS)[10] to examine the role of NER in the biocompatibility of click-linked DNA in human cells. Unlike MCF-7 cells however, XP2OS cells are SV40 transformed, which results in the replication of the click-linked plasmid prior to its transcription.[11] A pentanucleotide sequence (GAGGC) has been shown to be necessary for the function of the SV40 origin of replication (SV40^ori^) and deletion of this sequence destroys the origin of replication.[12] We therefore used a PCR-mediated plasmid-DNA deletion method[13] to remove this sequence from the pmCherry plasmid to give the ΔSV40^ori^-pmCherry plasmid; 63±1 % of the 244 cells injected with this ΔSV40^ori^-pmCherry plasmid displayed the red fluorescence associated with correct expression of mCherry (Figure 3 a, b). This plasmid was used as the template for the generation of click-linked and canonical pmCherry plasmid as above; 66±4 % of the 263 cells injected with the product of SDM with canonical mutagenic primers, and 60±3 % of the 283 cells injected with the click-linked plasmid expressed mCherry (Figure 3 a, b). The observed similarity in the percentage of cells that expressed mCherry from the canonical and click-linked plasmids demonstrates that NER is not required for biocompatibility and that our triazole-linked DNA is truly biocompatible with the transcriptional machinery of human cells.

**Figure 3:**
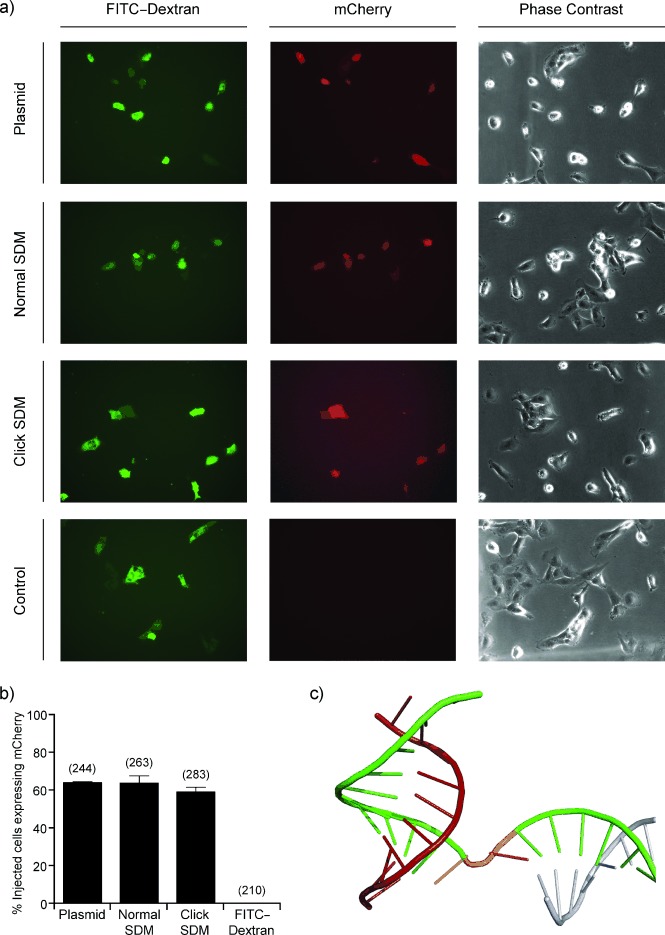
Click-linked DNA is functional in NER-deficient XP2OS cells. a) Representative images of cells injected with the ΔSV40^ori^-pmCherry plasmid or the products of SDM (with ΔSV40^ori^-pmCherry as template) with canonical primers, click-linked mutagenic primers, or water control. Injected cells are readily identifiable through the green fluorescence of the coinjected FITC–dextran dye. Those cells expressing mCherry from the injected plasmids can be identified by their red fluorescence. The phase contrast channel shows the population of XP2OS cells. b) The percentage of injected cells showing red fluorescence. The presented data is the average of two independent repeats and the numbers in brackets above each column give the total number of cells injected. c) The structure of DNA bound to polymerase II (protein data bank 1Y1W) shows that the template strand (green) is twisted open (highlighted in orange). The nontemplate strand (blue) and the transcribed RNA (red) are also shown. RNA polymerase II residues are not shown.

The practical limit on the length of error-free oligonucleotide synthesis has necessitated the use of enzymes for the assembly of polynucleotide chains into genes.[14,15] However, these approaches have been constrained by the assumption that the phosphodiester backbone that links oligonucleotides is critical for the biocompatibility and cellular function of the resulting DNA. As demonstrated in this work, this is not the case. Our results strongly suggest that RNA polymerase II, the enzyme responsible for all mRNA synthesis in eukaryotes, correctly transcribes the genetic information contained on a click-linked strand of DNA. The structure of the *Saccharomyces cerevisiae* RNA polymerase II transcribing complex, which is highly homologous to its human equivalent, reveals an extensive series of interactions between the phosphodiester backbone of the template DNA strand and the enzyme.[16] The presence of the triazole linker likely partially reduces the strength of these electrostatic interactions as it passes through the enzyme, however, the modified nucleotide is only one of the thirteen nucleotides that interact with the enzyme complex at any given time, and it is therefore unlikely to cause a significant weakening of the affinity of the enzyme for the DNA. The triazole DNA linker also reduces the Gibbs free energy for the dissociation of the two base pairs on either side,[17] therefore the activation energy for strand separation and formation of the transcription bubble is likely reduced for bases adjacent to the triazole linker. However, as demonstrated above, these changes do not affect the fidelity of transcription through click-linked DNA. When bound to polymerase II, the template DNA strand is significantly distorted,[16a] bending at 100°–140° between the upstream and downstream elements of the elongation complex,[18] with the dinucleotide at this junction being substantially twisted (Figure 3 c). It appears that the triazole linker does not significantly prohibit the template strand from adopting this conformation, otherwise transcription would have stalled at the linker. The absence of stalled transcription products when reading through click-linked DNA with T7 RNA polymerase in vitro further supports this hypothesis.[19]

Our results indicate that a phosphodiester linker is not essential for joining oligonucleotides for gene synthesis and open up the possibility of replacing enzymatic ligation with highly efficient chemical reactions. This approach would not necessarily be limited to the linker reported here, and alternative chemical reactions and the resulting linkers may also be suitable for this purpose. As the biological significance of the wide array of epigenetic DNA modifications becomes apparent,[20] the need for a purely chemical approach to the synthesis of epigenetically modified genes and genomes increases. In light of this, we are currently developing chemical DNA-ligation methods for one-pot gene assembly.
