# Supplementary material for: Transcription of Click-Linked DNA in Human Cells
Source: Angew Chem Int Ed Engl. 2014 Jan 22;53(9):2362–5. doi: 10.1002/anie.201308691 (PMC4016740; doi:10.1002/anie.201308691)
Supplement: Supplementary file 1 [file anie0053-2362-sd1.pdf]

Angewandte Chemie
Eine Zeitschrift der Gesellschaft Deutscher Chemiker

Supporting Information
© Wiley-VCH 2014
69451 Weinheim, Germany

Transcription of Click-Linked DNA in Human Cells**

*Charles N. Birts, A. Pia Sanzone, Afaf H. El-Sagheer, Jeremy P. Blaydes, Tom Brown, and Ali Tavassoli**

anie_201308691_sm_miscellaneous_information.pdf

Supplemental Figures

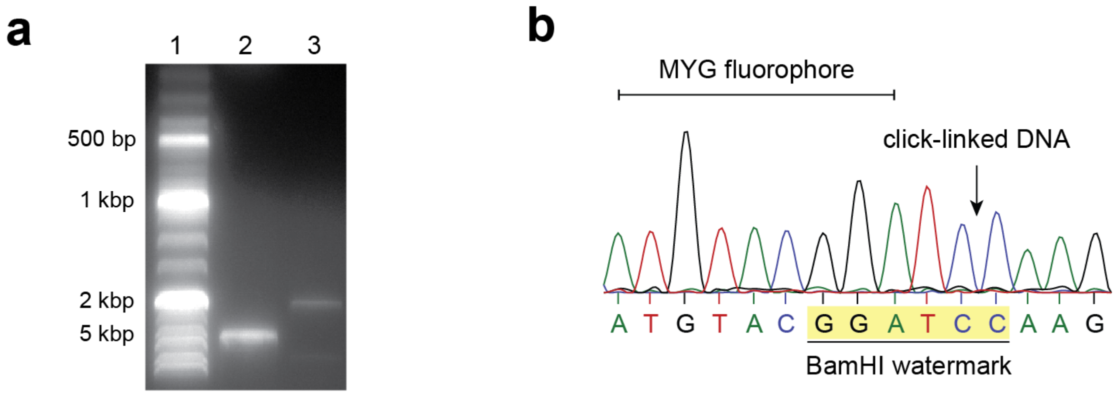

Figure S1. Verification of the click-linked pmCherry plasmid. a) Gel electrophoresis (0.8% agarose) of SDM reaction. Lane 1 is ladder; lane 2 is the product of SDM with the mutagenic click-linked primers after DpnI digestion (expected size 4696 bp); lane 3 is the template pmCherry plasmid, which migrates faster through the gel, as it is supercoiled. b) Representative sequencing data of the click-linked pmCherry plasmid. All examined cases showed the presence of the BamHI watermark associated with the click-linked DNA.

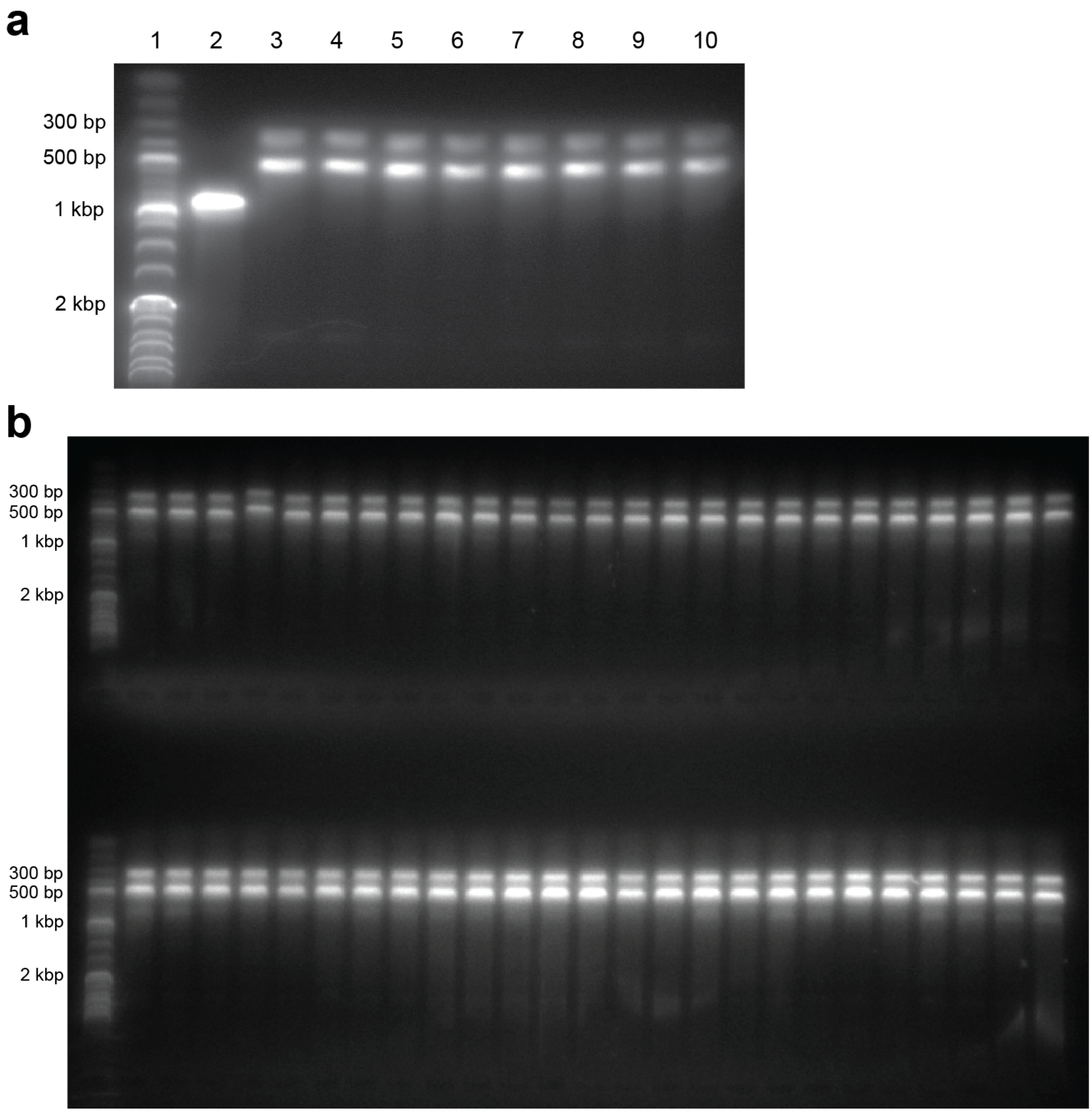

Figure S2. Representative restriction digestion of mCherry cDNA from microinjected MCF-7 cells. a) BamHI restriction digestion of the PCR product (amplifying the mCherry gene) of reverse transcribed mRNA from cells injected with click-linked pmCherry. Lane 1 is ladder, lane 2 is control showing digestion of the product of cells injected with canonical pmCherry (single band at 800 bp due to lack of BamHI watermark), lanes 3-10 are representative colonies containing the mCherry gene amplified from cDNA of cells injected with click-linked pmCherry (two bands at 300 bp and 500 bp due to presence of BamHI watermark). b) Representative digestion analysis of a further 50 colonies containing the mCherry gene amplified from cDNA of cells injected with click-linked pmCherry. Lane 1 in both rows is ladder. Lanes 2-26 in both rows are digestion analysis as detailed above. In all examined cases, the BamHI watermark was present as evident from two bands at 300 bp and 500 bp.

Methods

Assembly of click-linked pmCherry

Synthesis of click-linked primers and SDM were conducted as previously detailed.[1] The pmCherry plasmid was constructed for this study from pEGFP-N1 (GenBank #U55762).

Cell culture and microinjection

MCF-7 cells were maintained in DMEM (Invitrogen) supplemented with 10% fetal bovine serum (Autogen Bioclear), 100 U/ml penicillin, 100 μg/ml streptomycin and 2 mM L-glutamine (Invitrogen). XP2OS cells were maintained in MEM with non-essential amino acids (PAA) and supplemented as above. Microinjection was performed as previously described.[2] DNA was injected at 20 ng/μl in phosphate buffered saline. 70 kDa FITC-dextran (Sigma) was co-injected at 1 μg/μl. Cells were imaged 20 h post-injection using an Olympus IX81 microscope with heated stage using Olympus xcellence software. Statistical analysis was performed using Fisher's exact analysis of contingency tables (GraphPad Prism).

Reverse transcription of mRNA from cells

RNA extraction from injected cells was performed 24 h post-injection using RNeasy mini kit (Qiagen). On-column DNase digestion was carried out to digest any remaining injected plasmid DNA as per manufactures protocol. Reverse transcription of isolated mRNA was performed using M-MLV reverse transcriptase (Promega) as per manufactures protocol. The absence of pmCherry plasmid in the reverse transcribed mixture was confirmed by the absence of PCR product corresponding to an 800 bp region on the plasmid backbone, which was present in control PCR with pmCherry template.

Reference

[1] A. P. Sanzone, A. H. El-Sagheer, T. Brown, A. Tavassoli, *Nucleic Acids Res* **2012**, *40*, 10567-10575.

[2] C. N. Birts, L. M. Bergman, J. P. Blaydes, *Oncogene* **2011**, *30*, 1272-1280.
